# The bromodomain inhibitor JQ1+ reduces calcium-sensing receptor activity in pituitary cell lines

**DOI:** 10.1530/JME-21-0030

**Published:** 2021-07-05

**Authors:** Kate E Lines, Anna K Gluck, Supat Thongjuea, Chas Bountra, Rajesh V Thakker, Caroline M Gorvin

**Affiliations:** 1 Academic Endocrine Unit, Oxford Centre for Diabetes, Endocrinology and Metabolism, Radcliffe Department of Medicine, University of Oxford, Oxford,UK; 2 Centre for Computational Biology, MRC Weatherall Institute of Molecular Medicine, John Radcliffe Hospital, University of Oxford, Oxford, UK; 3 Centre for Medicines Discovery, Nuffield Department of Medicine, University of Oxford, Oxford, UK; 4 Institute of Metabolism and Systems Research and Centre for Endocrinology, Diabetes and Metabolism, University of Birmingham, Birmingham, UK; 5 Centre of Membrane Proteins and Receptors (COMPARE), University of Birmingham, Birmingham, UK

**Keywords:** corticotrophinoma, epigenetic modification, G protein-coupled receptor, pituitary tumourigenesis

## Abstract

Corticotrophinomas represent 10% of all surgically removed pituitary adenomas, however, current treatment options are often not effective, and there is a need for improved pharmacological treatments. Recently, JQ1+, a bromodomain inhibitor that promotes gene transcription by binding acetylated histone residues and recruiting transcriptional machinery, has been shown to reduce proliferation in a murine corticotroph cell line, AtT20. RNA-Seq analysis of AtT20 cells following treatment with JQ1+ identified the calcium-sensing receptor (CaSR) gene as significantly downregulated, which was subsequently confirmed using real-time PCR and Western blot analysis. CaSR is a G protein-coupled receptor that plays a central role in calcium homeostasis but can elicit non-calcitropic effects in multiple tissues, including the anterior pituitary where it helps regulate hormone secretion. However, in AtT20 cells, CaSR activates a tumour-specific cAMP pathway that promotes ACTH and PTHrP hypersecretion. We hypothesised that the *Casr* promoter may harbour binding sites for BET proteins, and using chromatin immunoprecipitation (ChIP)-sequencing demonstrated that the BET protein Brd3 binds to the promoter of the *Casr* gene. Assessment of CaSR signalling showed that JQ1+ significantly reduced Ca^2+^_e_-mediated increases in intracellular calcium (Ca^2+^_i_) mobilisation and cAMP signalling. However, the CaSR-negative allosteric modulator, NPS-2143, was unable to reduce AtT20 cell proliferation, indicating that reducing CaSR expression rather than activity is likely required to reduce pituitary cell proliferation. Thus, these studies demonstrate that reducing CaSR expression may be a viable option in the treatment of pituitary tumours. Moreover, current strategies to reduce CaSR activity, rather than protein expression for cancer treatments, may be ineffective.

## Introduction

Pituitary tumours are common neoplasms, which account for 10–15% of primary intracranial tumours and are identified in > 25% of unselected autopsies and approximately 20% of the population undergoing intracranial imaging (Daly *et al.* 2009, Di Ieva *et al.* 2014). Corticotrophinomas, which secrete adrenocorticotropic hormone (ACTH), represent 10% of all surgically removed pituitary adenomas (Daly *et al.* 2009). The recommended treatment for corticotrophinomas is trans-sphenoidal resection, which can result in remission rates of up to 90% for microadenomas (Cuevas-Ramos *et al.* 2016). Trans-sphenoidal resection can, however, result in mortality rates of 1–2%, with 10-year recurrence rates of ~20% (Daly *et al.* 2009, Cuevas-Ramos *et al.* 2016). Pharmacological treatments, including inhibitors of steroidogenesis, glucocorticoid antagonists, dopamine agonists, and somatostatin analogues, may provide an alternative for patients in whom surgery is contraindicated or has been unsuccessful (Cuevas-Ramos *et al.* 2016). However, these current medical treatments are often not effective in corticotrophinomas, and, therefore, there is a clinically unmet need for improved pharmacological treatments for these patients.

Increasing evidence indicates that aberrant epigenetic modifications play an important role in pituitary tumourigenesis (Shariq & Lines 2019), and previously we have demonstrated that the bromo and extra terminal domain (BET) protein inhibitor, JQ1+, reduces proliferation and increases apoptosis of the corticotroph murine cell line, AtT20 (Lines *et al.* 2020), indicating it may also represent an effective novel therapy for corticotrophinomas. The mechanism by which JQ1+ alters proliferation and apoptosis in these cells is, however, incompletely understood. To elucidate the target pathways of JQ1+ in pituitary cells, we previously undertook RNA sequence analysis (Lines *et al.* 2020). This revealed that one of the most highly significantly downregulated genes was the calcium-sensing receptor (CaSR) (Supplementary Table 1, see section on supplementary materials given at the end of this article).

The CaSR is a G protein-coupled receptor (GPCR) that is widely expressed and has calcitropic roles, that is, regulation of extracellular calcium (Ca^2+^_e_) by the parathyroid, kidneys and bone, and non-calcitropic roles such as inflammation, bronchoconstriction, wound healing, gastropancreatic hormone secretion, hypertension, and glucose metabolism (Hofer *et al.* 2000, Rossol *et al.* 2012, Yarova *et al.* 2015, Zietek & Daniel 2015). On stimulation by elevations in Ca^2+^_e_, the CaSR can couple to multiple G-protein subtypes to activate diverse signalling pathways. In most cell types, CaSR couples predominantly to G_q__/11_, to activate phospholipase C (PLC)-mediated increases in intracellular calcium and mitogen-activated protein kinase (MAPK), and G_i__/o_, to reduce cAMP (Brown & MacLeod 2001, Hofer & Brown 2003). However, in some cell types, CaSR is able to couple to G_12/13_ to activate Rho kinase signalling (Huang *et al.* 2004), and in breast cancer and AtT20 cells, CaSR activates G_s_-mediated increases in cAMP (Mamillapalli *et al.* 2008, Mamillapalli & Wysolmerski 2010). The switching of CaSR coupling from G_q__/11_ and G_i__/o_ to G_s_ has been suggested to contribute to tumourigenesis as it is only present in malignant breast cancer cells and not in normal mammary epithelial cells (Mamillapalli *et al.* 2008). This switch to G_s_ coupling increases cAMP, which consequently increases secretion of parathyroid hormone-related peptide (PTHrP) (Mamillapalli *et al.* 2008), a growth factor that is required for mammary gland development (Hens *et al.* 2007) but has been implicated in malignancy in several tissue types including breast, colon and prostate (Suva *et al.* 1987, Asadi *et al.* 1996, Shen *et al.* 2007). Targeting this pathway by inhibiting CaSR signalling via treatment with the CaSR negative allosteric modulator NPS-2143, and mammary gland-specific deletion of the *Casr* gene, reduced tumour cell proliferation and PTHrP secretion (Kim *et al.* 2016).

A CaSR-mediated increase in cAMP signalling and PTHrP secretion via the G_s_ pathway has also been demonstrated in AtT20 cells (Mamillapalli & Wysolmerski 2010). Reduction of CaSR expression by siRNA has been shown to reduce CaSR signalling in AtT20 cells, although the effect on proliferation was not assessed (Mamillapalli & Wysolmerski 2010). Recently, JQ1+ treatment of AtT20 cells was shown to reduce proliferation and increase apoptosis, indicating that this may be an efficacious treatment for corticotrophinomas (Lines *et al.* 2020). RNA-Seq analysis in these JQ1+-treated cells revealed that the *Casr* gene was significantly downregulated (Lines *et al.* 2020) (Supplementary Table 1). We, therefore, hypothesised that reducing CaSR expression or signalling by the receptor may be a novel treatment for pituitary tumours. Moreover, if existing allosteric modulators of CaSR could reduce AtT20 proliferation, these compounds could be repurposed for use in pituitary tumours.

## Materials and methods

### Cell lines, antibodies and compounds

AtT20 murine pituitary corticotroph tumour cells (ATCC) were cultured in DMEM media (Gibco, supplemented with 10% foetal calf serum (FCS) (Gibco). HEK293 cells stably expressing CaSR (HEK-CaSR) (Nesbit *et al.* 2013) were cultured in DMEM Glutamax media (Gibco), supplemented with 10% FCS and 400 µg/mL geneticin (ThermoFisher Scientific). All cells were maintained at 37°C, 5% (vol/vol) CO_2_, and tested for mycoplasma using the MycoAlert kit (Lonza, Basel, Switzerland). Cells were maintained for a maximum of 15 passages, and all assays were performed after at least one passage following cell rederivation. Cells were transiently transfected with Lipofectamine 2000 (LifeTechnologies). The active (+)-JQ1 (JQ1+, IUPAC [(S)-4-(4-chloro-phenyl)-2,3,9-trimethyl-6H-1-thia-5,7,8,9a-tetraaza-cyclopenta[e]azulen-6-yl]-acetic acid tert-butyl ester) and inactive control compound (−)-JQ1 (JQ1−) were supplied by the Structural Genomics Consortium, Oxford, and further details on the structure and specificity for each compound are available at https://www.thesgc.org/chemical-probes. Most compounds were suspended/diluted in DMSO (Sigma-Aldrich), except pertussis toxin (Sigma), which was suspended in ethanol. JQ1+ was used at a concentration of 1 µM, which has previously been shown to reduce AtT20 cell proliferation (Lines *et al.* 2020). The negative allosteric modulator NPS-2143 hydrochloride (also known as 2-chloro-6-[(2R)-3-[[1,1-dimethyl-2-(2-naphthalenyl)ethyl]amino]-2-hydroxypropoxy]-benzonitrile hydrochloride) was obtained from Sigma-Aldrich (catalogue SML0362). H-89 dihydrochloride (H89) was purchased from Sigma-Aldrich. Untreated and DMSO or ethanol only-treated cells were used as controls.

### Quantitative real time PCR (qRT-PCR)

Total RNA was extracted from AtT20 cell lines using a miRvana kit (Ambion), and 1 μg was used to generate cDNA using the Quantitect RT kit (Qiagen), as previously described (Lines *et al.* 2017). Quantitect primers (Qiagen) were used for qRT-PCR reactions, utilising the Quantitect SYBR green kit (Qiagen), on a Rotor-Gene 5. Each test sample was normalised to the geometric mean of reference genes for glyceraldehyde-3-phosphate dehydrogenase and α1a-tubulin (*Gapdh* and *Tuba1a*, respectively). The relative expression of target cDNA in all qRT-PCR studies was determined using the Pfaffl method (Pfaffl 2001). Data were normalised to untreated cells (set at 1) within each biological replicate, then data were combined for all the biological replicates to perform statistical analyses. Statistical analysis was performed using either a one-way ANOVA or an unpaired student’s *t*-test.

### Western blot analysis

Western blots were performed as previously described (Lines *et al.* 2017, Gorvin *et al.* 2018). Cells were seeded at 300,000 cells/well, left to settle for 24 h before treatment with 1 µM JQ1−, JQ1+, or DMSO. Cell lysates were prepared in NP40 lysis buffer: 250 mM NaCl, Tris 50 mM (pH 8.0), 5 mM EDTA, 0.5% NP-40 (vol/vol) and 2× protease inhibitor tablets (Roche). Lysates were prepared in 4× Laemmli loading dye (BioRad Laboratories) boiled at 95°C for 5 min, resolved using 6% SDS-PAGE gel electrophoresis and transferred to polyvinylidene difluoride membrane (PVDF). PVDF membrane was probed with primary antibody anti-CaSR (ADD, Abcam) and secondary antibody anti-mouse HRP conjugate (BioRad). Blots were then stripped with Restore Western Blot Stripping Buffer (ThermoScientific) and re-probed with anti-Calnexin (Millipore) loading control primary antibody and secondary antibody anti-rabbit HRP conjugate (Santa Cruz Biotechnology). Western blots were visualised using an Immuno-Star WesternC kit (BioRad) on a BioRad Chemidoc XRS+ system. Densitometry analysis was performed by calculating the number of pixels per band using ImageJ software. Data were represented as the number of pixels of the protein band, relative to the number of pixels of the corresponding calnexin band. Western blot analyses were performed in four biological replicates (i.e. four independently treated batches of cells, prepared on separate days). To compare datasets, data were normalised to untreated cells (set at 1) within each biological replicate, then data were combined for all the biological replicates to perform statistical analyses. Statistical analysis was performed using an unpaired student’s *t*-test.

### Chromatin immunoprecipitation and sequencing (ChIP-Seq)

Approximately 8 million AtT20 cells were fixed in 1% formalin in culture media before being split into four aliquots and sonicated to produce DNA fragments between 200 and 1000 bp. Chromatin Immunoprecipitation (ChIP) was undertaken using the ChIP assay kit (Merck Millipore) with samples incubated overnight with 10 µL anti-Brd2 (D89B4, Cell Signalling), 10 µL anti-Brd3 (61489, Active motif, La Hulpe, Belgium), and 0.5 µg anti-Brd4 (PA5-41550, Thermo Fisher Scientific) antibodies. DNA from each ChIP was un-cross-linked, purified using phenol extraction, precipitated using ethanol and resuspended in nuclease-free water. For all experiments, an input (no antibody) control was also included for analysis. ChIPs were sequenced using a NovaSeq 6000 at the Oxford Genomics Centre (Wellcome Centre for Human Genetics, University of Oxford). All experiments were performed in *n*  = 3 biological replicates. For analysis, adapter filtering, read mapping and filtering, lane merging, de-duplication, peak calling and refining were undertaken, as well as normalized strand coefficient (NSC), relative strand correlation (RSC), irreproducible discovery rate (IDR), read enrichment around transcription start site (TSS) quality checks. BigWig files were generated using the ‘bamCoverage’ function from deepTools2 software (Ramirez *et al.* 2016). The reads per genomic content (RPGC) (1× normalisation) approach was used for the coverage normalisation with parameters applied as follows: bin Size 10 – normalise using RPGC – effective GenomeSize 2620345972 – extend reads – ignore duplicates. Data were then uploaded and visualised using a UCSC genome browser custom track.

### Intracellular calcium measurements

Ca^2+^_e_-induced Ca^2+^_i_ responses were measured by Fluo-4 calcium assays, as previously described (Gorvin *et al.* 2018). Cells were plated at 30,000 cells/well in black-walled 96-well plates (Corning). Cells were incubated with serum-free media (SFM) overnight with either vehicle (DMSO), JQ1− or JQ1+ (Fluo-4 dye was prepared according to manufacturer’s instructions (Invitrogen), and cells were loaded for 1 h at 37°C. Baseline measurements were made and increasing concentrations of CaCl_2_ were injected automatically into each well. Changes in Ca^2+^_i_ were recorded on a PHERAstar instrument (BMG Labtech, Aylesbury, UK) at 37°C with an excitation filter of 485 nm and an emission filter of 520 nm. The peak mean fluorescence ratio of the transient response after each individual stimulus was expressed as a normalised response, relative to the basal response. Nonlinear regression of concentration-response curves, area under the curve (AUC) and maximal responses were calculated using GraphPad Prism 7. Each treatment was performed in four to five columns on four separate occasions. Maximal responses were compared using one-way ANOVA and EC_50_ values compared using the F-test (Gorvin *et al.* 2018).

### Luciferase reporter assays

Cells were plated at 50,000 cells/well in 24-well plates and transiently transfected with 100 ng/mL pGL4-cAMP-response element (CRE) luciferase reporter and 10 ng/mL pRL null control luciferase reporter constructs (Promega). For control experiments, HEK-CaSR cells were pre-treated with 10 μM forskolin (MP Biomedicals, Eschwege, Germany) for 30 min on the day of the assay to activate adenylate cyclase. For studies with epigenetic modifiers, cells were pre-treated with DMSO vehicle control, JQ1+ or JQ1− compounds for 24 h prior to performance of assays, and for allosteric modulator studies cells were exposed to 20 nM NPS-2143 overnight. On the day of the experiment, all cells were treated with SFM containing 0.1–10 mM CaCl_2_ and incubated for 4 h. Cells were then lysed and assays were performed using Dual-Glo Luciferase (Promega) on a Veritas Luminometer (Promega) as previously described (Gorvin *et al.* 2018). For studies with pertussis toxin, cells were pre-incubated with ethanol vehicle control or 300 ng/mL pertussis toxin for 6 h prior to CRE measurements. For studies with H89, cells were pre-incubated with DMSO vehicle control or 10 µM H89 for 1 h prior to luciferase measurements. Luciferase: renilla ratios were expressed as fold changes relative to responses at low CaCl_2_ concentrations (0.1 mM). All assay conditions were performed in four independent transfections. Statistical analysis was performed by two-way ANOVA with Tukey’s multiple-comparisons test using GraphPad Prism 7.

### Cell viability

Cell viability was assessed using the CellTiter-Blue Cell Viability assay (Promega), as previously described (Lines *et al.* 2017). Cells were plated at 10,000 cells/well and grown in media containing 1.8 mM CaCl_2_ (basal level for DMEM-Glutamax media). For each assay, 20 μL of CellTiter-Blue reagent were added per well, incubated for 2 h at 37°C, 5% (vol/vol) CO_2_, before the output was read on a CytoFluor microplate reader (PerSeptive Biosystems, Paisley, UK) at 530 nm excitation and 580 nm emission. All assay conditions were performed in four to five biological replicates. Statistical analyses were performed by one-way ANOVA and Student’s *t*-test using GraphPad Prism 7.

### Statistical analysis

Statistical comparisons in qRT-PCR and Western blot analyses were made using one-way ANOVA or the unpaired student’s *t*-test. For Ca^2+^_i_ measurements, maximal responses were compared using one-way ANOVA and EC_50_ values were compared using the F-test. For luciferase reporter assays, statistical analysis was performed by two-way ANOVA with Tukey’s multiple-comparisons test using GraphPad Prism 7. For cell viability assays, statistical analysis was performed by one-way ANOVA and Student’s *t*-test using GraphPad Prism 7.

## Results

### CaSR expression is significantly downregulated by JQ1+ treatment

**Figure 1 Calcium-sensing receptor:**
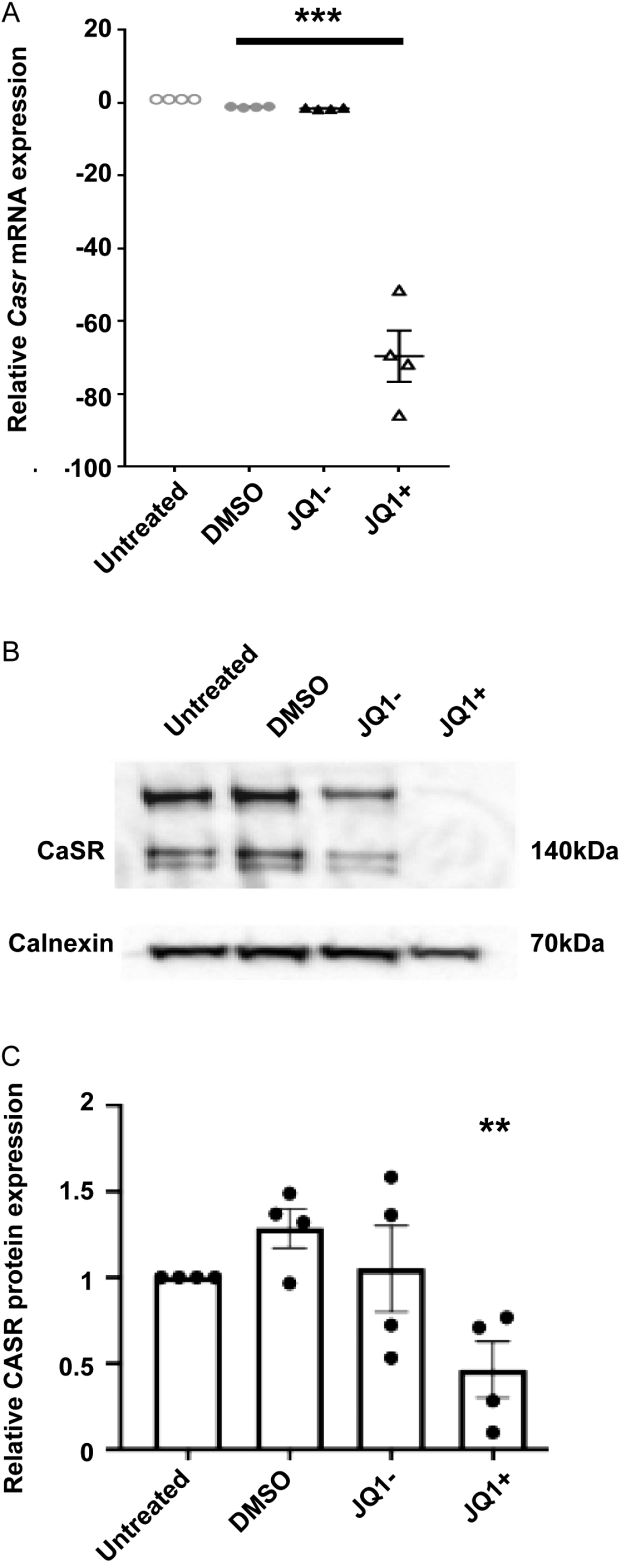
(CaSR) is downregulated by JQ1+ treatment. (A) qRT-PCR analysis of *CaSR* following exposure to cell culture media, DMSO, JQ1− and JQ1+ for 96 h in AtT20 cells. (B) Representative Western blot of CaSR in AtT20 cells following exposure to DMSO, JQ1− and JQ1+ for 96 h. (C) Densitometry analysis of protein expression following treatment with DMSO, JQ1− and JQ1+ for 96 h. CaSR protein expression was expressed relative to calnexin. Data are expressed as mean ± s.e.m. in panels A and C. Statistical analyses compared to DMSO-treated cells in all panels: ***P < 0.001, **P < 0.01.

Our previous studies demonstrated that JQ1+ was effective in reducing proliferation in the corticotrophinoma cell line, AtT20 (Lines *et al.* 2020). RNA-sequencing showed a number of genes to be differentially expressed in AtT20 cells by JQ1+ treatment, including the gene encoding *Casr*, which was in the top 15 most downregulated genes (43.30-fold reduction compared to vehicle-treated cells, *P* < 0.0001) (Supplementary Table 1). Quantitative RT-PCR analysis was used to verify the reduction in *Casr* gene expression in JQ1+-treated cells. This revealed a significant reduction in JQ1+-treated cells (−69.67 ± 7.07-fold, *P* < 0.001) compared to untreated cells (Fig. 1A). There was no significant difference between AtT20 cells treated with DMSO or JQ1− when compared to untreated cells (−1.12 ± 0.08 and −1.50 ± 0.12, respectively), indicating the downregulation observed with JQ1+ is specific to the active JQ1+ compound (Fig. 1A). Western blot analysis confirmed that CaSR protein expression was also reduced in JQ1+-treated AtT20 cells (−0.46 ± 0.16), compared to untreated cells (Fig. 1B and C ). There was no significant difference between AtT20 cells treated with DMSO or JQ1− when compared to untreated cells (1.29 ± 0.11 and 1.05 ± 0.25), respectively. Therefore, JQ1+ treatment reduces mRNA and protein expression of CaSR in AtT20 cells.

### The epigenetic modifier Brd3 binds to DNA regions coding for **Casr**

JQ1+ is an epigenetic modifier that targets bromodomains (BRDs) of the BET protein family, which in turn bind acetylated histone residues (Filippakopoulos *et al.* 2010). The BET family comprises four members, BRD2, BRD3, BRD4, which are ubiquitously expressed, and BRDT, a testes-specific protein (Filippakopoulos *et al.* 2010). Our previous studies have shown that Brd2, Brd3 and Brd4 are expressed at high levels in the AtT20 cells (Lines *et al.* 2020), and JQ1+ may target any of these proteins. We, therefore, performed ChIP-Seq analysis, which can be used to identify the binding sites of DNA-associated proteins (Park 2009), using antibodies targeting each of the three Brd proteins in AtT20 cell lines. This revealed that ChIP with Brd3 antibody resulted in significant enrichment (*P* < 0.001), compared to input control, of DNA in the genetic region coding for the *Casr* (Supplementary Fig. 1). In total, two peaks were observed, the first at an average position of 16:3,653,074–36,532,125, and the second at 16:3,655,597–36,556,077, both of which correspond to regions within intron 1–2 of the mouse *Casr* gene ENSMUG00000051980. This significant enrichment was not observed for the ChIPs with Brd2 or Brd4 antibodies (Supplementary Fig. 1), thereby indicating that Brd3 likely binds to genetic regions coding for the *Casr*, while Brd2 and Brd4 do not bind or bind to a lesser extent.

### JQ1+ inhibits G_s_-mediated cAMP increases

**Figure 2:**
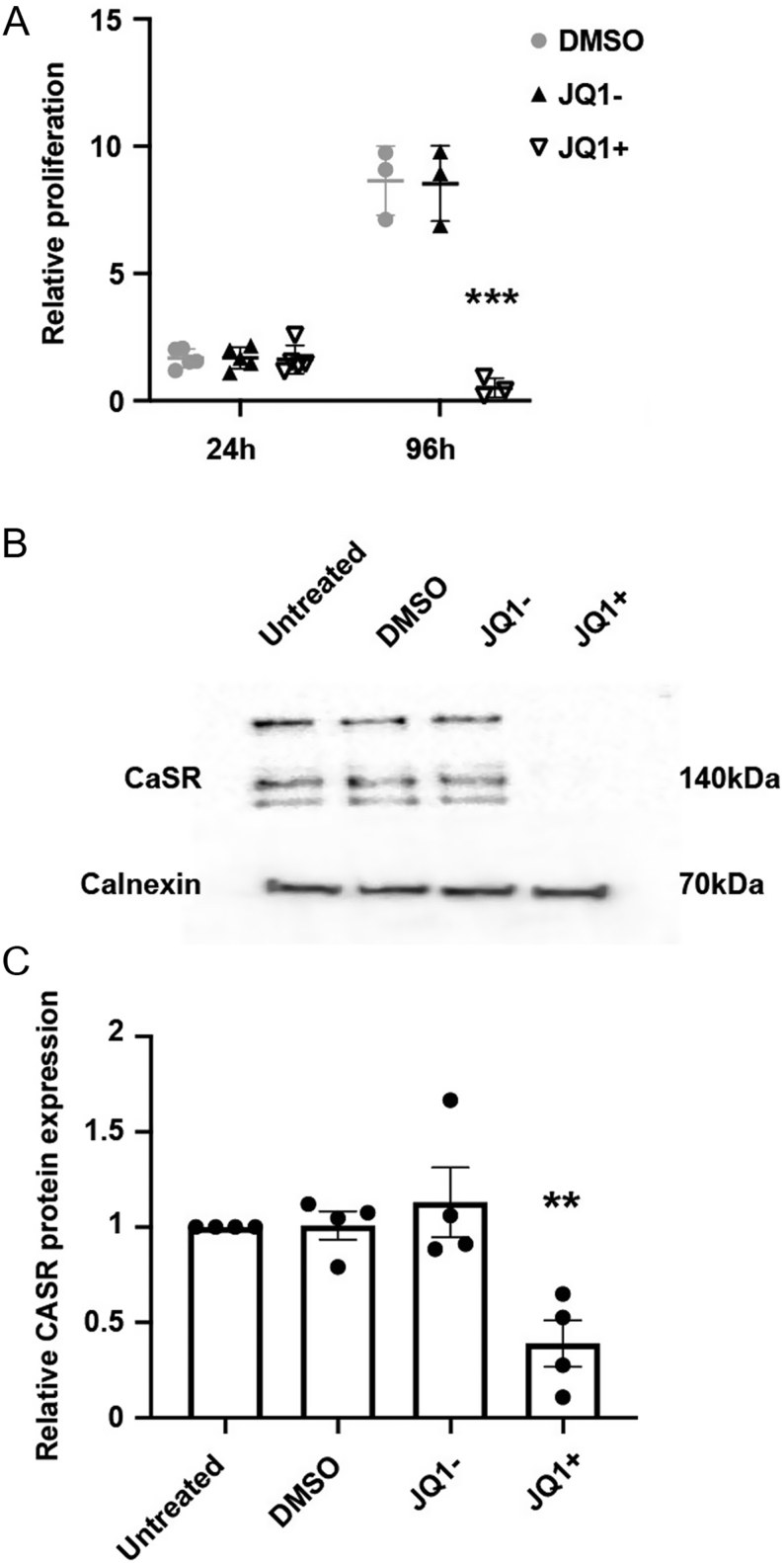
JQ1+ does not reduce proliferation of AtT20 cells after 24 h but does decrease calcium-sensing receptor (CaSR) protein expression. (A) Relative proliferation of AtT20 cells following exposure to DMSO, JQ1− and JQ1+ for 24 and 96 h. Data were expressed relative to cell numbers at day 0. (B) Representative Western blot of CaSR in AtT20 cells following exposure to DMSO, JQ1− and JQ1+ for 24 h. (C) Densitometry analysis of protein expression following treatment with DMSO, JQ1− and JQ1+ for 24 h. CaSR protein expression was expressed relative to calnexin. Data are expressed as mean ± s.e.m. in panels A and C. Statistical analyses compared to DMSO treatment in panel A, ***P < 0.001, and statistical analysis compared to DMSO-treated cells in panel C, **P < 0.005.

As JQ1+ reduced CaSR expression in AtT20 cells, we hypothesised that CaSR signalling would also be reduced. However, previous studies have shown that JQ1+ reduces the number of viable AtT20 cells after 96-h treatment, which is at least in part due to increased apoptosis (Lines *et al.* 2020). Therefore, any reduction in signalling could be due to a JQ1+-mediated impairment of signalling or merely a reduction in cell number. Thus, prior to embarking on signalling studies, we first sought to establish a time point at which JQ1+ would not reduce proliferation but may still impact CaSR function. To assess the number of viable cells following treatment with DMSO, JQ1+ and JQ1−, Cell Titer Blue assays were performed 24 and 96 h post-treatment (Fig. 2A). This revealed a modest increase in cell numbers in all treatment groups after 24 h, with no significant difference between JQ1+ and the other treatment groups (Fig. 2A). In contrast, at 96 h, cells treated with DMSO and JQ1− exhibited a 5.2-fold and 5.1-fold increase in cell number, respectively, while JQ1+ cells reduced cell viability by 17-fold (*P* < 0.005, Fig. 2A). This confirmed previous findings that JQ1+ reduced viability in AtT20 cells at 96 h and revealed that the compound did not significantly reduce cell numbers at 24 h. Moreover, JQ1+ treatment significantly reduced CaSR protein expression at 24 h, by 61.2% (*P* < 0.005, Fig. 2B and C ). There was no significant difference between CaSR expression in cells treated with DMSO or the inactive JQ1− compound when compared to untreated cells. Thus, we concluded that 24 h post-JQ1+ treatment would be the optimal time to perform signalling assays.

**Figure 3:**
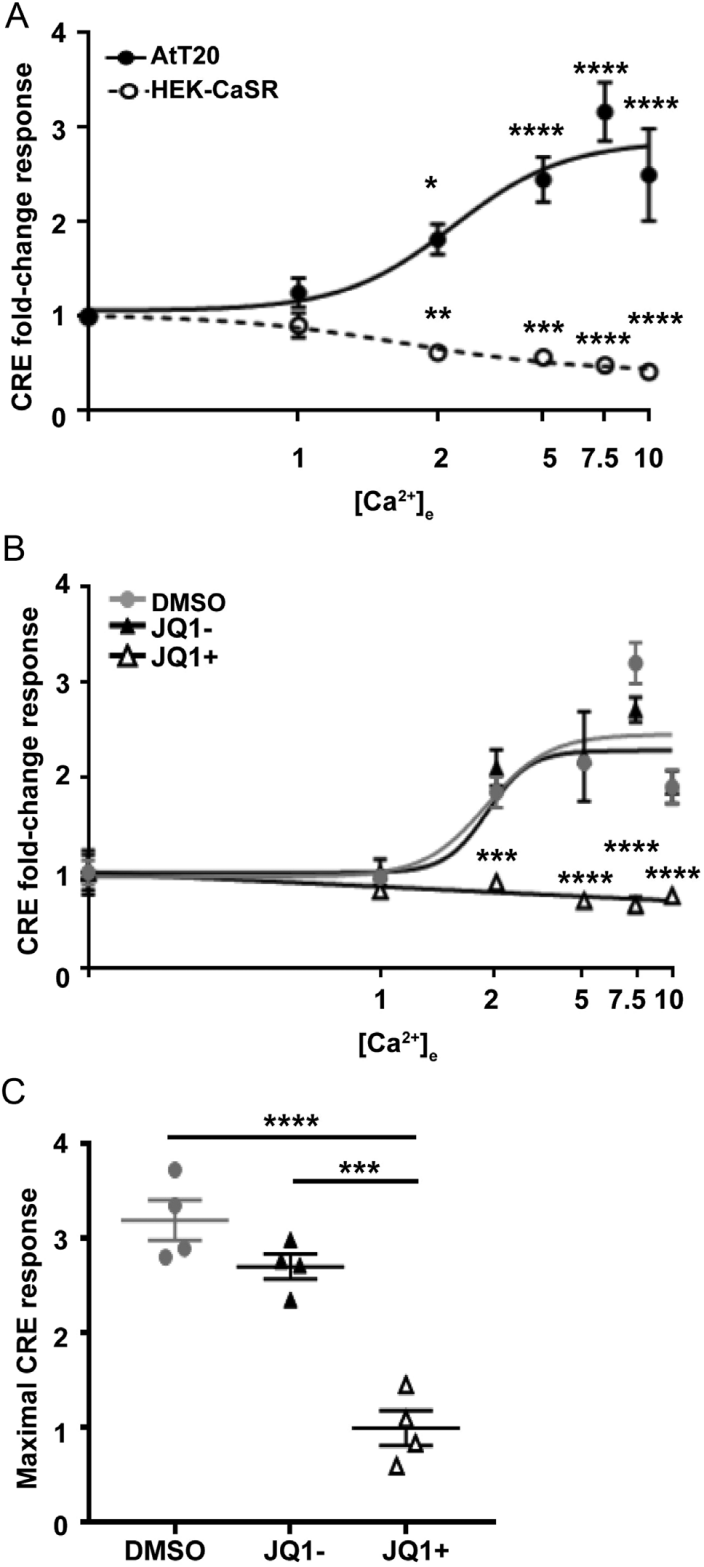
JQ1+ reduces cAMP signalling in AtT20 cell lines. (A) Ca^2+^_e_-induced cAMP-response element (CRE) luciferase reporter responses in AtT20 and HEK-CaSR cell lines. Elevations in Ca^2+^_e_ concentrations increase CRE luciferase in AtT20 cells and reduce luciferase in HEK-CaSR cells. (B) Ca^2+^_e_-induced CRE luciferase reporter responses in AtT20 cell lines following exposure to DMSO, JQ1− and JQ1+ for 24 h. (C) Maximal CRE luciferase responses from panel B. Data are expressed as mean ± s.e.m. in all panels. Statistical analyses compared to basal responses in each cell line in panel A and to DMSO-treated cells in panel B. Firefly values ranged from 6027 to 981,659 luminescent units and renilla from 1027 to 5031 luminescent units. ****P < 0.0001, ***P < 0.001, **P < 0.01, *P < 0.05.

Previous studies of CaSR in cancer cell lines, including MCF-7 human breast cancer cells and AtT20 cells, have shown that CaSR switches from preferentially coupling to G_q__/11_ and G_i__/o_ pathways to exclusively signalling by a G_s_ pathway (Mamillapalli *et al.* 2008, Mamillapalli & Wysolmerski 2010). Activation of the G_s_ signalling pathway leads to a signalling cascade involving activation of adenylate cyclase, increases in cAMP, activation of protein kinase A, and increases in transcription (Walsh & Van Patten 1994). We used a CRE luciferase reporter (Gorvin *et al.* 2018) to assess cAMP signalling. Although CRE can be induced by other signalling pathways (e.g. Ca^2+^_i_ and MAPK), CaSR-mediated effects on CRE luciferase are impaired by pertussis toxin (which inhibits adenylate cyclase) and H89 (which impairs protein kinase A) in HEK-CaSR cells, indicating that CaSR-mediated CRE luciferase activity is at least partially cAMP-dependent (Supplementary Fig. 2). We thus utilised this reporter in the AtT20 cells to first confirm that Ca^2+^_e_ preferentially activates CRE luciferase activity (Fig. 3). AtT20 cells were exposed to increasing concentrations of Ca^2+^_e_ (0.1–10 mM), and CRE reporter activity was measured. This revealed a concentration-dependent increase in CRE reporter activity, with a maximal stimulatory fold-change response (E_max_) of 3.35 ± 0.29, which was significantly elevated compared to cells exposed to 0.1 mM Ca^2+^_e_ (*P* < 0.0001) (Fig. 3A). The same concentration-response assay was also performed in HEK-CaSR cells. This showed a significant reduction in luciferase reporter activity in a concentration-dependent manner, as previously reported (Gorvin *et al.* 2018) (maximal inhibitory response of 0.68 ± 0.06, *P* < 0.0001 compared to 0.1 mM Ca^2+^_e_). This reduction in signalling is most likely due to G_i__/o_-coupled effects on cAMP rather than pERK or MAPK as these latter two pathways are increased by CaSR rather than reduced (Fig. 3A). Thus, these studies confirm that AtT20 cells preferentially couple to G_s_ pathways, while HEK-CaSR cells couple to G_i__/o_ pathways.

To determine whether JQ1+ affected CaSR-mediated cAMP responses, we repeated the CRE luciferase reporter assays following overnight treatment with DMSO control, JQ1+ or JQ1− (Fig. 3B, C and D ). JQ1+ abolished the Ca^2+^_e_-induced increase in CRE reporter activity, while vehicle and JQ1− cells exhibited a concentration-dependent increase in CRE reporter activity (Fig. 3C). Thus, the maximal responses in DMSO and JQ1−-treated cells were 3.20 ± 0.21-fold and 2.71 ± 0.13-fold, respectively, whereas in JQ1+-treated cells, the maximal response was 1.00 ± 0.18-fold, observed in cells treated with basal calcium (0.1 mM) (Fig. 3C). Therefore, JQ1+ is effective in reducing CaSR G_s_-mediated signalling in AtT20 cells.

### JQ1+ inhibits calcium mobilisation in AtT20 cells

**Figure 4:**
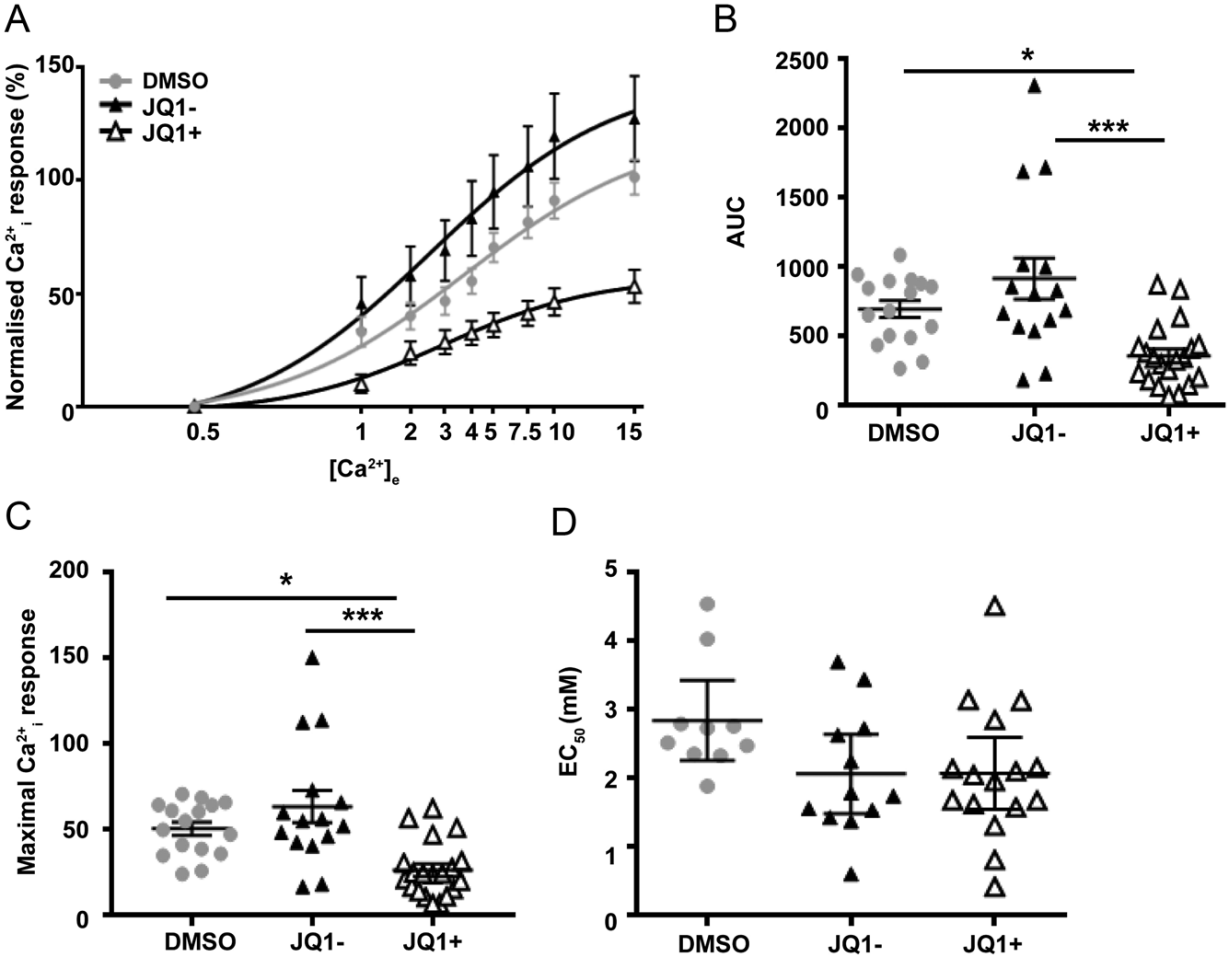
JQ1+ reduces calcium-sensing receptor (CaSR)-mediated calcium mobilisation in AtT20 cell lines. (A) Ca^2+^_e_-induced Fluo-4 intracellular calcium mobilisation assays in AtT20 cells following exposure to DMSO, JQ1− and JQ1+ for 24 h. (B) Area under the curve (AUC) of data in A. (C) Maximal Ca^2+^_i_ responses and (D) EC_50_ values obtained in Fluo-4 intracellular calcium mobilisation assays shown in panel A. Data are expressed as mean ± s.e.m. in panels A and B, and mean ± 95% CIs in panel C. Statistical analyses compared between groups in panel B, ***P < 0.001, *P < 0.05.

Previous studies of AtT20 cells have demonstrated that CaSR can also activate Ca^2+^_i_ signalling pathways (Emanuel *et al.* 1996). The effects of DMSO, JQ1+ and JQ1− on Ca^2+^_e_-induced Ca^2+^_i_ responses in AtT20 cells were assessed using the Fluo-4 calcium assay. The Ca^2+^_i_ responses were shown to be elevated in a dose-dependent manner following stimulation with increasing concentrations of Ca^2+^_e_ (Fig. 4A). The responses of the AtT20 cells treated with DMSO or JQ1− were similar at all concentrations of calcium, and the maximal responses were not significantly different (101.4 ± 7.73 and 127.3 ± 18.84, respectively) (Fig. 4A, B and C ). In contrast, the JQ1+-treated cells had reduced responses compared to both DMSO and JQ1−-treated cells (E_max_ = 53.15 ± 7.27) (Fig. 4A, B and C ). Despite the reduction in Ca^2+^_i_ maximal responses, there was no significant difference between the EC_50_ values of the three treatment groups (Fig. 4D). This indicates that the potency of AtT20 for Ca^2+^_e_ is unchanged, while the efficacy is reduced, most likely due to the reduced CaSR protein expression.

### Treatment with a CaSR negative allosteric modulator reduces CaSR signalling but does not affect proliferation in AtT20 cells

**Figure 5:**
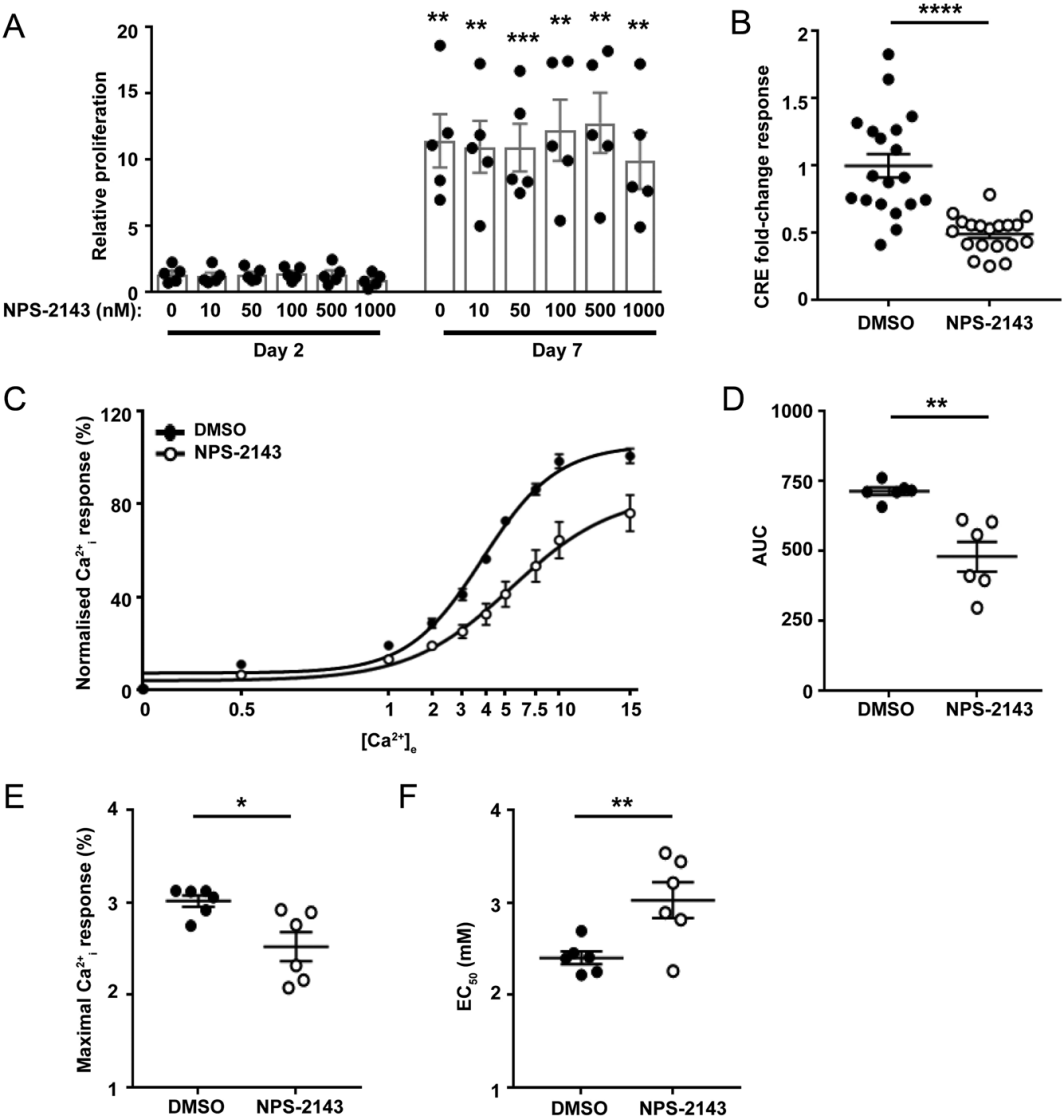
NPS-2143 has no effect on AtT20 proliferation but does reduce calcium-sensing receptor (CaSR)-mediated cAMP and Ca^2+^_i_ signalling in AtT20 cells. (A) Effect of NPS-2143 (0–1000 nM) on proliferation of AtT20 cells 2 days and 7 days post-treatment. Data were expressed relative to proliferation at day 1. Statistical analyses comparing proliferation between day 2 and day 7 in each group. There was no significant difference between cell proliferation in cells exposed to different concentrations of NPS-2143. Raw fluorescence units ranged between 17,096 and 69,941 for day 2 and 19,935 and 222,377 for day 7. (B) cAMP-response element (CRE) luciferase reporter responses at 5 mM Ca^2+^_e_ in AtT20 cells exposed to DMSO or 20 nM NPS-2143 for 12 h. Data were expressed relative to responses in DMSO-treated cells. (C) Ca^2+^_e_-induced Fluo-4 intracellular calcium mobilisation assays in AtT20 cells following exposure to DMSO or 20 nM NPS-2143. Raw fluorescence units ranged between 1896 and 58,337. (D) Area under the curve (AUC) of data in C, (E) Maximal Ca^2+^_i_ responses, and (F) EC_50_ values showing mean ± 95% CIs obtained in Fluo-4 intracellular calcium mobilisation assays shown in panel C. ****P < 0.0001, ***P < 0.001, **P < 0.01, *P < 0.05.

These studies led us to hypothesise that JQ1+ most likely mediates its effects by a reduction in CaSR protein rather than mediating direct effects on signalling pathways. To test whether reducing CaSR signalling led to changes in proliferation, we assessed the effect of the CaSR-negative allosteric modulator NPS-2143 on AtT20 cell viability over 7 days. Cells were exposed to six different concentrations of NPS-2143 (0, 10, 50, 100, 500 and 1000 nM) and compared to untreated cells (Fig. 5). As exposure of cells to high concentrations of calcium over a period of days is likely to have a detrimental effect on cells, we maintained cells in standard DMEM media, which contains 1.8 mM CaCl_2_. Proliferation increased in all cells over 7 days, however, NPS-2143 did not have a significant effect on cell number at any concentration tested (Fig. 5A). In contrast, 50 nM NPS-2143 still reduced CaSR signalling in AtT20 cells, demonstrated by a reduction in CRE luciferase reporter activity (Fig. 5B). NPS-2143 also reduced Ca^2+^_e_-mediated Ca^2+^_i_ signalling demonstrated by a rightward shift in the concentration-response curve when compared to DMSO-treated cells and reduced AUC (Fig. 5B, C and D ) and a reduced maximal response (E_max_ = 100.63 ± 3.13 in vehicle compared to 75.99 ± 7.76 in NPS-2143-treated cells) and an increased mean EC_50_ value of 3.12 mM (95% (CI) 2.79–3.49) in NPS-2143-treated cells compared to 2.38 mM (95% CI 2.23–2.53) in DMSO-treated cells (Fig. 5E and F ). Thus, reducing CaSR signalling alone is not sufficient to decrease proliferation in AtT20 cells.

## Discussion

Our studies showed that JQ1+ treatment of corticotroph pituitary cells impairs CaSR mRNA and protein expression (Fig. 1), which may contribute to the reduced cell proliferation observed in earlier studies (Lines *et al.* 2020). This indicates that targeting CaSR expression could be beneficial in reducing proliferation in neuroendocrine tumours, such as corticotrophinomas that represent up to 10% of all surgically removed pituitary adenomas (Daly *et al.* 2009), however, this remains to be tested in detail. Moreover, studies of CaSR function in anterior pituitary cells have shown that CaSR activation may enhance pituitary hormone secretions, including ACTH and PTHrP from AtT20 cells (Ferry *et al.* 1997, Mamillapalli & Wysolmerski 2010); growth hormone (GH) secretion from rat somatotrophs (Zivadinovic *et al.* 2002); and enhancement of GH-releasing hormone (GHRH)-mediated GH secretion in human non-functioning pituitary adenoma and GH-omas (Romoli *et al.* 1999). Thus, decreasing CaSR expression may reduce the hypersecretion of ACTH and GH that are observed in 4.8–10% and 13–20% of pituitary adenomas, respectively, and could provide a possible treatment for Cushing’s disease and acromegaly, common co-morbidities of ACTH and GH hypersecretion (Mehta & Lonser 2017, Lines *et al.* 2020). However, compounds intended to reduce CaSR protein expression would need to be specifically targeted to pituitary cells to prevent adverse effects, such as severe hypercalcaemia that would be observed if CaSR expression was reduced in parathyroid glands and kidneys, in which CaSR performs its primary roles in calcium homeostasis.

The observation that the JQ1+ compound abolished CaSR activity and cell proliferation in AtT20 cells led us to hypothesise that reducing CaSR signalling by treatment with a negative allosteric modulator, NPS-2143, could also reduce cell proliferation and may be a novel treatment for corticotrophinomas. However, while 20 nM NPS-2143 was effective at reducing both Ca^2+^_i_ and cAMP signalling in AtT20 cells, it had no effect on pituitary cell proliferation in our assays (Fig. 5). Moreover, higher concentrations of NPS-2143 (up to 1000 nM) had no significant effect on AtT20 cell proliferation (Fig. 5). This contrasts with previous studies that have shown NPS-2143 to be efficacious in reducing proliferation in bone metastases associated with renal cell carcinoma (Joeckel *et al.* 2014), breast cancer cell lines (Kim *et al.* 2016), gastric cancer cell lines (Zhang *et al.* 2020), melanoma M14 cancer cells (Wang *et al.* 2018) and human prostate PC-3 cells (Yamamura *et al.* 2019). However, these studies used a higher dose of the negative allosteric modulator than our studies, ranging from 5 to 100µM. These concentrations are far in excess of that required to maximally inhibit CaSR activity in HEK293 cells (IC_50_ = 43 nM) (Gowen *et al.* 2000) and to stimulate PTH secretion from bovine parathyroid cells *in vitro* (EC50 = 39 nM) (Nemeth *et al.* 2001). Indeed, when lower concentrations of NPS-2143 were tested in some of these cancer cell lines, the compound was ineffective in reducing proliferation (Wang *et al.* 2018, Zhang *et al.* 2020). The findings from these previous studies indicate that high doses of NPS-2143 that reduce CaSR expression, as well as activity, may be required for NPS-2143 to be effective in reducing the growth of cancer cells. However, this remains to be investigated in detail.

Several lines of evidence indicate that a reduction in CaSR expression, rather than decreasing CaSR activity, is required to reduce cell proliferation. Exposure of cells to high doses of NPS-2143 (10 µM) reduces CaSR protein expression (Huang & Breitwieser 2007, Yamamura *et al.* 2019), indicating it is likely this, rather than a reduction in CaSR activity, that is responsible for the reduced proliferation observed in multiple cancer cell lines. Treatment of breast cancer cells with siRNAs that target the CaSR reduced receptor expression and decreased cell proliferation (Kim *et al.* 2016). Finally, our studies using JQ1+ downregulated CaSR mRNA and protein expression (Fig. 1) and reduced AtT20 cell viability. Although JQ1+ was also effective in reducing CaSR signalling (Figs 3 and 4), it is likely that this is due to the reduced CaSR protein expression, which was apparent at 24-h post-treatment with JQ1+. Thus, these studies indicate that the current strategy to reduce CaSR activity in several tumour models may be ineffective unless higher concentrations of compounds are used that can reduce CaSR protein expression.

Previous studies have shown that treatment of AtT20 pituitary cells with JQ1+ alters the expression of other genes that have a role in proliferation, including nuclear factor κ-light-chain enhancer of activated B cells (NFκB) and somatostatin receptor 2 (SSTR2) (Lines *et al.* 2020). However, the effect of this reduced expression on NFκB and SSTR2 activity was not investigated, and it remains to be determined whether signalling by these proteins is impaired in AtT20 cells. In the present study, we show that *Casr* is also downregulated by JQ1+ in AtT20 cells, and that this results in reduced CaSR activity. Moreover, the effect of the other genes downregulated in JQ1+-treated AtT20 cells (Supplementary Table 1) remains to be investigated in detail. Downregulation of the keratin 23 (*Krt23*) gene has been shown to reduce proliferation in colon cancer cells (Birkenkamp-Demtroder *et al.* 2013), although its function has not been explored in pituitary cells. Other genes, such as LIM homeobox transcription factor 1α (*Lmx1a*), have been associated with cancer but this appears to act as a tumour suppressor (Liu *et al.* 2009), and thus the effect of its downregulation by JQ1+ on the growth of AtT20 cells is unknown. Further investigation of these genes could reveal novel targets for the treatment of corticotrophinomas. It is possible that the observed reduction in proliferation and increased apoptosis of AtT20 cells following treatment with JQ1+ is due to the combined effect of changes in multiple proteins, including NFκB, SSTR2 and CaSR. Further studies investigating the effects of BET inhibition on each of these pathways would be required to determine whether all three pathways contribute to the effects of AtT20 cell growth.

Our data indicate that Brd3 was enriched in the genetic region coding for the CaSR*,* and, therefore, may play a role in the regulation of *Casr* expression (Supplementary Fig. 1). BRD3 is a BET family member that binds acetylated histones and facilitates transcription by recruitment of other proteins (Filippakopoulos *et al.* 2010, Fujisawa & Filippakopoulos 2017). The downstream targets of BRD3 in the pituitary, and indeed in cancers in general, are not well described, with BRD3 mostly associated with the regulation of erythroid target genes after binding of GATA1 (Lamonica *et al.* 2011, Stonestrom *et al.* 2015). The role of BRD3 in calcium regulation has also not been investigated in detail. However, BRD3 has been reported to be a calcium-sensitive gene that modulates transcriptional machinery in response to calcium signalling during *Xenopus* renal organ development (Bibonne *et al.* 2013). It is, therefore, plausible that a feedback loop could be present between calcium signalling and BRD3 expression, which ultimately can downregulate *Casr* expression.

In conclusion, our studies demonstrate that the BET inhibitor JQ1+ impairs CaSR mRNA and protein expression that may contribute to the reduction in cell proliferation observed in AtT20 corticotroph pituitary cells. Thus, reducing CaSR expression may be a viable option in the treatment of pituitary tumours. Moreover, we have shown that reducing CaSR activity, but not protein expression, is unlikely to have a major effect on pituitary tumourigenesis.

## Supplementary Material

Supplementary Table 1 The 10 most highly downregulated genes in JQ1+ versus JQ- treated AtT20 cells, as determined by RNA sequencing

Supplementary Figure 1 Brd3 binding is enriched in the genomic region of the CaSR The UCSC genome browser of ChIP-Seq tracks of AtT20 cells using antibodies for Brd2 (shown in red), Brd3 (shown in blue) and Brd4 (shown in orange). The higher read coverage of Brd3 at the Casr gene is observed compared to ChIPs with no antibody input control (shown in black). Raw data and input subtracted data displayed is a representative image from n=3 biological replicates. The scale bar represents 50kb.

Supplementary Figure 2 CRE luciferase responses are reduced by pertussis toxin and H89 Assessment of CaSR-mediated CRE luciferase reporter activity in HEK-CaSR cells treated with (A) pertussis toxin (PTx), which inhibits adenylate cyclase activity, or (B) H89, which inhibits protein kinase A. Data is expressed as mean±SEM in all panels. Statistical analyses comparing responses to vehicle treated cells. ****p<0.0001.

## Declaration of interest

The authors declare that there is no conflict of interest that could be perceived as prejudicing the impartiality of the research reported.

## Funding

This work was supported by an Early Career Grant from the Society for Endocrinology http://dx.doi.org/10.13039/501100000382 (K E L, C M G); a Wellcome Trust http://dx.doi.org/10.13039/100010269 Senior Investigator Award (grant number 106995/Z/15/Z) (R V T) and the Horizon 2020 Programme of the European Union (Project ID: 675228) (R V T).

## Author contribution statement

K E L and C M G designed the study. K E L, A K G, S T and C M G performed the experiments and analysed the data. K E L and C M G wrote the manuscript. C B and R V T provided the materials. K E L, A K G, S T, C B, R V T and C M G reviewed and edited the manuscript.
