# Supplementary material for: The bromodomain inhibitor JQ1+ reduces calcium-sensing receptor activity in pituitary cell lines
Source: J Mol Endocrinol. 2021 Jul 5;67(3):83–94. doi: 10.1530/JME-21-0030 (PMC8345903; doi:10.1530/JME-21-0030)
Supplement: Supplementary Table 1 The 10 most highly downregulated genes in JQ1+ versus JQ- treated AtT20 cells, as determined by RNA sequencing [file supplementary_table_1.pdf]

Supplementary Table 1 The 10 most highly downregulated genes in JQ1+ versus JQ-treated AtT20 cells, as determined by RNA sequencing

Gene	Adjusted p-value	Fold change
Kif12	6.8933E-129	-670.85
Apoa5	8.9985E-18	-163.36
Lmx1a	2.96026E-17	-148.89
Dio2	3.9329E-208	-78.20
Gm3693	5.30346E-21	-57.44
Gm26512	2.14834E-34	-52.41
Krt23	0.00E+00	-51.62
Mycs	2.21968E-59	-50.42
Mgat5b	9.99312E-42	-50.18
Kcnj11	3.37611E-50	-48.30
Samd3	2.18071E-12	-45.05
Casr	**2.7488E-178**	**-43.30**
Adamtsl2	4.7203E-212	-40.72
Gsdma	2.893E-192	-40.14
Tcea3	3.84935E-14	-38.77
R3hdml	2.72895E-18	-38.74
E530001K10Rik	6.86106E-14	-37.17
Trp73os	1.78098E-13	-35.56
Th	0.00E+00	-35.28
Slc16a14	2.13928E-33	-33.95
