# Supplementary figures and images for: The bromodomain inhibitor JQ1+ reduces calcium-sensing receptor activity in pituitary cell lines
Source: J Mol Endocrinol. 2021 Jul 5;67(3):83–94. doi: 10.1530/JME-21-0030 (PMC8345903; doi:10.1530/JME-21-0030)

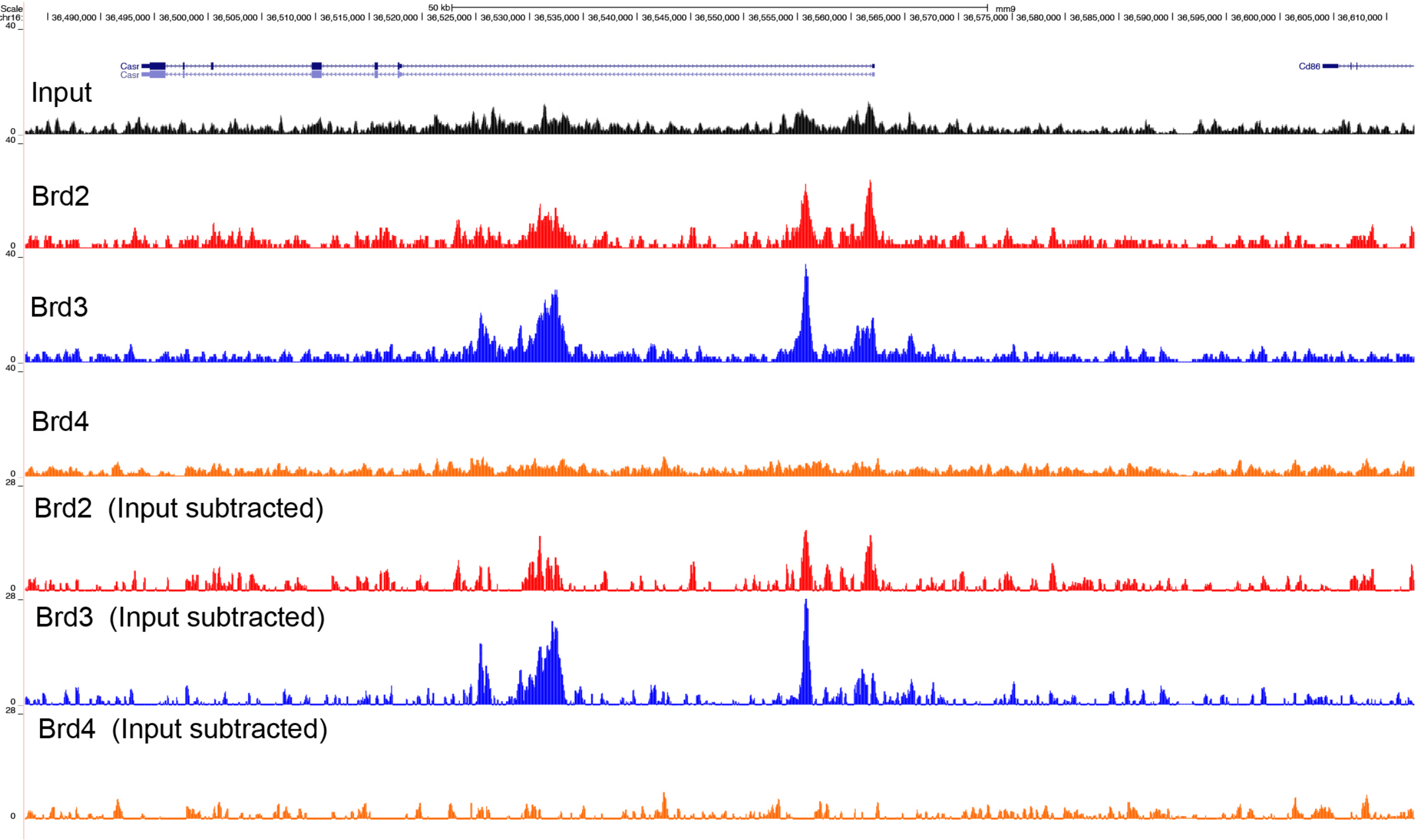

Supplement: Supplementary Figure 1 Brd3 binding is enriched in the genomic region of the CaSR The UCSC genome browser of ChIP-Seq tracks of AtT20 cells using antibodies for Brd2 (shown in red), Brd3 (shown in blue) and Brd4 (shown in orange). The higher read coverage of Brd3 at the Casr gene is observed compare [file supplementary_figure_1.pdf]

**A**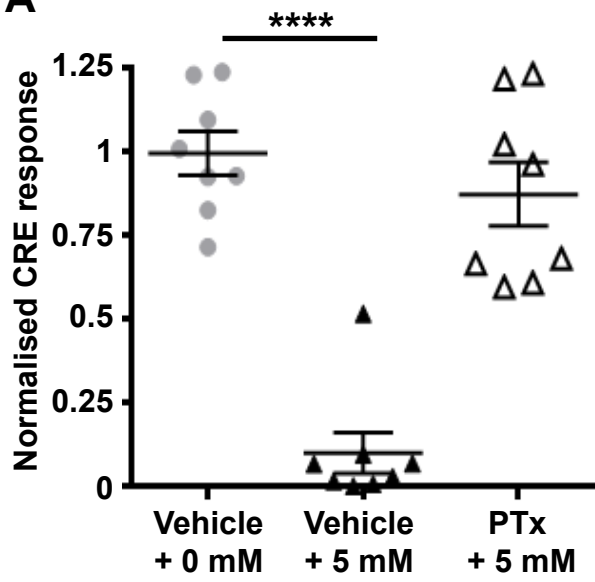**B**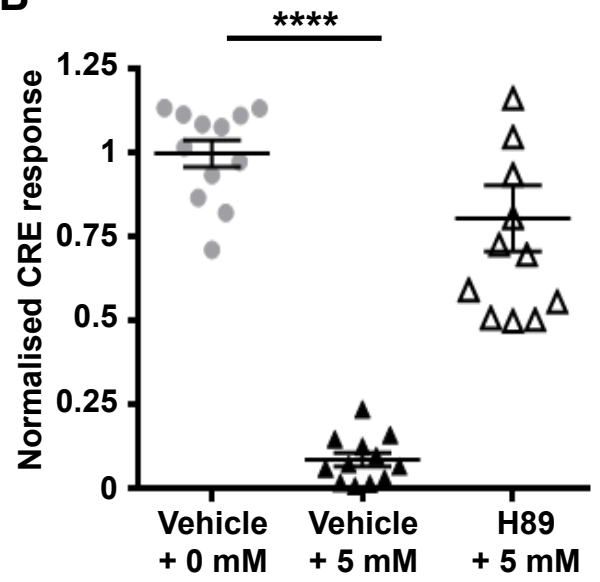

Supplement: Supplementary Figure 2 CRE luciferase responses are reduced by pertussis toxin and H89 Assessment of CaSR-mediated CRE luciferase reporter activity in HEK-CaSR cells treated with (A) pertussis toxin (PTx), which inhibits adenylate cyclase activity, or (B) H89, which inhibits protein kinase A. Data i [file supplementary_figure_2.pdf]
